# Identification of Novel Proteins in *Neospora caninum* Using an Organelle Purification and Monoclonal Antibody Approach

**DOI:** 10.1371/journal.pone.0018383

**Published:** 2011-04-04

**Authors:** Catherine S. Sohn, Tim T. Cheng, Michael L. Drummond, Eric D. Peng, Sarah J. Vermont, Dong Xia, Stephen J. Cheng, Jonathan M. Wastling, Peter J. Bradley

**Affiliations:** 1 Department of Microbiology, Immunology and Molecular Genetics, University of California Los Angeles, Los Angeles, California, United States of America; 2 Division of Laboratory Animal Medicine, University of California Los Angeles, Los Angeles, California, United States of America; 3 Department of Infection Biology, Institute of Infection and Global Health, University of Liverpool, Liverpool, United Kingdom; Institut national de la santé et de la recherche médicale - Institut Cochin, France

## Abstract

*Neospora caninum* is an important veterinary pathogen that causes abortion in cattle and neuromuscular disease in dogs. *Neospora* has also generated substantial interest because it is an extremely close relative of the human pathogen *Toxoplasma gondii*, yet does not appear to infect humans. While for *Toxoplasma* there are a wide array of molecular tools and reagents available for experimental investigation, relatively few reagents exist for *Neospora*. To investigate the unique biological features of this parasite and exploit the recent sequencing of its genome, we have used an organelle isolation and monoclonal antibody approach to identify novel organellar proteins and develop a wide array of probes for subcellular localization. We raised a panel of forty-six monoclonal antibodies that detect proteins from the rhoptries, micronemes, dense granules, inner membrane complex, apicoplast, mitochondrion and parasite surface. A subset of the proteins was identified by immunoprecipitation and mass spectrometry and reveal that we have identified and localized many of the key proteins involved in invasion and host interaction in *Neospora*. In addition, we identified novel secretory proteins not previously studied in any apicomplexan parasite. Thus, this organellar monoclonal antibody approach not only greatly enhances the tools available for *Neospora* cell biology, but also identifies novel components of the unique biological characteristics of this important veterinary pathogen.

## Introduction

*Neospora caninum* is an obligate intracellular parasite in the phylum Apicomplexa that infects a large number of mammals and causes disease in dogs and cattle [1], [2], [3]. *Neospora* is closely related to *Toxoplasma gondii*, an important human pathogen of immunocompromised patients and neonates [4]. Both *Neospora* and *Toxoplasma* can invade and proliferate *in vitro* in every nucleated mammalian cell type tested and also infect a wide array of mammals [3], [5]. Remarkably, while *Toxoplasma* infects as much as a third of the human population worldwide and causes severe disease in immunocompromised patients and neonates, *Neospora* does not appear to infect humans [1], [4], [6]. This key difference in host range of these highly similar parasites emphasizes the importance of comparative analyses of the two organisms, which are currently in progress using genomic, transcriptomic, and proteomic approaches [5],[6],[7],[8].

*Neospora* and *Toxoplasma* are extremely similar in many aspects of the lytic cycle of the tachyzoite form of the parasites [6], [9]. Both parasites first attach loosely to their host cells in events that are likely mediated by a family of highly abundant GPI-anchored surface antigens [6]. The micronemes then release a common series of molecular adhesins onto the surface of the parasite which further mediate attachment and engage the parasite's actin:myosin motor to provide the driving force for host cell invasion [10]. Next, the rhoptries are released to form the “moving junction,” a tight region of contact between the invading parasite and the host cell [11]. The ring-like moving junction appears to serve two functions: first, as a scaffold for the parasite to grip the host cell for entry and second, as a filter of host transmembrane proteins from the nascent vacuole, thereby enabling entry and avoidance of subsequent fusion with host lysosomes. The rhoptries also inject a series of proteins into the cytosol of the host that modulate host cell functions, ensuring an optimal infection [12]. Finally, the dense granules secrete proteins that further modify the vacuole for intracellular survival [13], [14], [15]. Both parasites replicate within similar membrane bound vacuoles and ultimately egress from the host cells upon which another lytic cycle is initiated. Intracellular replication is dependent on many processes, but the parasite mitochondrion and apicoplast are key subcellular organelles whose biosynthetic pathways are known targets for therapeutic intervention [16], [17],[18].

While these processes are highly similar in general, a closer examination of the invasive processes has highlighted some significant differences between *Neospora* and *Toxoplasma*. For example, while the surface of both parasites is dominated by a family of GPI-anchored surface antigens, glycosylation of the surface proteins between the two parasites appears to be substantially different as assessed by dye and lectin staining [19]. This suggests that these parasite surface molecules may differ in how they mediate the initial interaction with the host. Similarly, the parasites attach to host cell glycosaminoglycans with differing affinities; *Toxoplasma* prefers heparin sulfate whereas *Neospora* prefers chondroitin sulfate, indicating that differences also exist in the host components that mediate the initial interaction [20]. Additional dissimilarities are revealed by the differential impact of various protease inhibitors on invasion, which likely reflects differences in the maturation of micronemal adhesins or rhoptry proteins secreted at the onset of invasion [21]. More differences are certain to emerge as our understanding of the players involved in the invasion process improves for both parasite systems.

Because *Toxoplasma* infects a large percentage of the human population and causes substantial morbidity and mortality in humans worldwide, a far greater amount is known at the molecular and cellular level regarding how this parasite infects its hosts. A wide array of tools has been developed for the study of *Toxoplasma* including genomic and proteomic data, microarrays, selectable markers, polyclonal and monoclonal antibodies, regulatable promoter systems, and a substantial number of knockout strains [9], [22], [23]. With the recent sequencing of the *N. caninum* genome (www.genedb.org/Homepage/Ncaninum and www.EuPathDB.org) a comparative analysis of the genomes is likely to reveal a large number of candidate proteins that may confer host specificity. Testing these candidates will undoubtedly require substantial new tools such as antibodies in *Neospora*, of which few are currently available. To aid in this effort and to identify novel proteins involved in *Neospora* infections, we raised a panel of monoclonal antibodies (mAbs) against a mixed organellar fraction of *N. caninum*. In this work, we obtained a variety of mAbs against a number of compartments in *Neospora* including the micronemes, rhoptries (body and neck), dense granules, mitochondrion, apicoplast, inner membrane complex, and parasite surface. Analysis of several of these antibodies revealed that we were able to obtain specific probes for some of the central players in parasite invasion including the *Neospora* orthologues of AMA1, RON4, and ROP2 family proteins. In addition, we were able to identify novel secreted proteins not previously localized in any system, thus expanding our understanding beyond what has already been defined in *Toxoplasma* and related apicomplexans.

## Materials and Methods

### Ethics Statement

Antibodies raised in mice were performed under the guidelines of the Animal Welfare Act and the PHS Policy on Humane Care and Use of Laboratory Animals. Specific details of our protocol were approved by the Chancellor's Animal Research Committee at University of California at Los Angeles (ARC# 2004-055).

### Parasite and host cell culture

NC1 strain *Neospora caninum* was grown and maintained by serial passage in confluent monolayers of human foreskin fibroblasts grown in DMEM media supplemented with 10% fetal calf serum plus penicillin, streptomycin and glutamine.

### Subcellular fractionation

A mixed fraction of *N. caninum* organelles was purified using a Percoll density gradient essentially as described for *T. gondii* [24]. 5×10^9^ *N. caninum* extracellular parasites were collected by centrifugation at 1200 *g* for 10 minutes at 25°C. All subsequent steps were carried out at 4°C. The parasites were washed once in PBS and once in R Buffer (10 mM MOPS pH 7.2, 250 mM sucrose, 2 mM DTT, 1 mM EDTA, 1X Roche Complete Protease Inhibitor Cocktail). The parasites were resuspended in R buffer at 5×10^8^ parasites/ml and were disrupted in the French Press as described. Intact parasites and large debris were removed by centrifugation at 1200 *g* for 15 minutes. The supernatant was then centrifuged at 25,000 *g* for 25 minutes to pellet the organelles. The organellar pellet was resuspended in R buffer plus 30% Percoll and centrifuged for 25 minutes at 61,500 *g*. An ∼2 ml fraction immediately above the rhoptry/dense granule band was collected. In *T. gondii*, this fraction corresponds to a mixed organellar fraction in consisting of rhoptries, micronemes, dense granules, apicoplasts, and mitochondria [24], [25]. To remove contaminating Percoll, the purified organelles were diluted to 10 ml in R buffer, and pelleted at 100,000 *g*. The organellar fraction was resuspended in R buffer and stored at −80°C until use.

### Monoclonal antibody production

The organellar fraction was used to immunize a single BALB/c mouse (∼300 µg per injection) on a 21-day immunization schedule. Five days after the 5th boost, the animal was euthanized and the spleen was collected for the fusion. The fusion was carried out using polyethylene glycol 4000 essentially as described [26], using P3X myeloma cells as the fusion partner and selecting for hybridomas in HAT medium. The resulting undiluted hybridoma supernatants were screened by immunofluorescence assay (IFA) in 2 sets of 96 well plates containing HFF's infected with *N. caninum* that had been fixed with either 100% methanol for 3 minutes or 3.7% formaldehyde for 15 minutes. Cells from positive wells were rescreened on three subsequent passages by IFA, and the positives were frozen in liquid nitrogen. Most of the positive hybridomas were further cloned by limiting dilution and rescreened as above.

### Immunofluorescence microscopy

For staining fixed parasites with the antibodies secreted from hybridomas, culture supernatants were collected and used undiluted on intracellular *N. caninum* essentially as described for similar assays in *T. gondii* [27], [28]. Colocalization was performed with MitoTracker Red (Invitrogen) for the mitochondria [29], Hoechst stain (Invitrogen) for the apicoplast [25], anti-MIC2 (1∶1000) for the micronemes [30], and anti-VSG (1∶10,000) for the rhoptries (using *N. caninum* tranfected with a ROP1-VSG targeting construct) [31]. The secondary antibodies used were 488-conjugated goat anti-mouse (1∶2000) and 594-conjugated goat anti-rabbit (1∶2000) (Invitrogen). The microscopy and imaging were performed as previously described [32], [33], [34].

### Western blot analysis

Western blots were performed using whole cell lysates of extracellular *N. caninum* tachyzoites under reducing conditions probed with undiluted tissue culture supernatants. Those that failed under reducing conditions were attempted under non-reducing conditions and the non-reducing conditions were only reported when the reduced conditions failed or had poor reactivity relative to non-reducing conditions (Table 1). The relative molecular weight (*M*r) is reported only when a single predominant band could be identified as the likely target band (Table 1).

**Table 1:** Characteristics of *Neospora* monoclonal antibodies.

mAb	Target site determined by IFA	IFA fixation conditions	M_R_ (kDa) determined by Western blot	Blot conditions
4C1	Surface	F	62	R
21H12	Surface	F	16	R
8H12	IMC	F	43	R
15D5	IMC	M	36	R
15G6	IMC	M	>250	R
3D9	Mitochondrion	F	33	R
4G10	Mitochondrion	M	33	R
8E10	Mitochondrion	F	17, 40	R
9G5	Mitochondrion	F	33, 60	R
14H8	Mitochondrion	F	UTD	
10C7	Apicoplast	F	60	R
10D8	Apicoplast	M	60	R
2D9	Rhoptry	F	36	R
6A4	Rhoptry	F	80	R
11F1	Rhoptry	F	60	R
11G3	Rhoptry	F	34	R
12D4	Rhoptry	M	65	R
16G4	Rhoptry	F	36	R
17E5	Rhoptry	F	36	R
18G9	Rhoptry	F	34	R
20B5	Rhoptry	F	65	R
20D2	Rhoptry	F	65	R
10G5	Rhoptry Neck	M	68	R
10H4	Rhoptry Neck	M	22, 62	NR
17H12	Rhoptry Neck	F	145	R
3A5	Vacuole	F	15	R
4B1	Vacuole	F	35	R
10B10	Vacuole	M	45	R
12C1	Vacuole	F	36	R
16B4	Vacuole	F	50	R
21H4B	Vacuole	F	40	R
4A4	Dense Granule	F	40	NR
21H7A	Dense Granule	F	20	R
3D12A	Microneme	F	72	R
10G6	Microneme	F	60	R
12F5	Microneme	F	UTD	
13A2	Microneme	F	35	NR
13C10	Microneme	F	UTD	
15G1	Microneme	F	36	R
16H9	Microneme	F	60	NR
18C2	Microneme	F	UTD	
21D2	Microneme	F	52	R
21G11	Microneme	F	36	R
21H8	Microneme	F	35	NR
14A4	Internal spots	F	32	R
17D4B	Internal spots	M	70	R

Abbreviations in the table: F: formaldehyde, M: methanol, NR: non-reduced, R: reduced,

UTD: unable to determine, IMC: inner membrane complex.

### Early invasion assays

Early invasion assays were performed similar to those previously described in *T. gondii* using a temperature shift [35]. Parasites were allowed to settle onto monolayers at 4°C, then briefly warmed for a time course of 2–10 minutes. The samples were then fixed and stained with mAbs that detected the rhoptry necks. Co-localization to the rhoptry necks and moving junction were determined by *T. gondii* RON4 antisera that cross-reacts with *N. caninum* diluted 1∶800 [25].

### Immunoaffinity purification of proteins for mass spectrometry

For immunoaffinity purification of *Neospora* proteins using the mAbs isolated, the antibodies were cross-linked to protein G sepharose (Sigma) as previously described [36]. Large-scale *Neospora* cultures were grown and 5×10^9^ extracellular parasites were lysed in radioimmunoprecipitation (RIPA) buffer plus complete protease inhibitor cocktail (Roche). Following binding, the column was washed 5 times with 10 ml RIPA buffer, and the proteins eluted with high pH (100 mM triethylamine [TEA] pH 11.5). The samples were dried in a speed-vac to remove the TEA and the samples resuspended in 1X sample buffer and loaded into a single well of 11 or 15% SDS-PAGE gels. The samples were stained with Coomassie Brilliant Blue R250 and the abundant band corresponding to the size of the protein detected by Western blot was excised and identified by mass spectrometry. In each case, the target band was easily identifiable and corresponded to the size of the protein detected by Western blot.

### Mass spectrometry identification of proteins

Proteins were identified by mass spectrometry as described [23]. Briefly, excised gel bands were destained in a solution of 50% (v/v) acetonitrile and 50 mM ammonium bicarbonate and cysteines reduced by incubation in 50 µl 10 mM DTT/100 mM ammonium bicarbonate, followed by alkylation in 50 µl 100 mM iodoacetamide/55 mM ammonium bicarbonate. Gel bands were then dehydrated with 100% (v/v) acetonitrile and then rehydrated with 25 µl 10 ng/µl sequencing grade trypsin/25 mM ammonium bicarbonate at 37°C before analysis by mass spectrometry.

### Reverse-phase high performance liquid chromatography and tandem mass spectrometry

LC MS/MS was carried out using an LTQ ion trap mass spectrometer (Thermo Fisher Scientific Inc) with an electrospray ionisation source, coupled downstream to an online nano pepMap100 c18 RP column (3 µm, 100 Å, 75 µm i.d. ×15 cm) on a Dionex Ultimate 3000 HPLC system (Dionex). A C18 trapping column (300 µm i.d. ×5 mm) desalted the peptides prior to their entry onto the analytical column, which was equilibrated with buffer comprising of water/2% (v/v) acetonitrile/0.1% (v/v) formic acid at a flow of 300 nl min^−1^. Tryptic peptides were eluted using a linear gradient of 0–50% (v/v) acetonitrile/0.1% (v/v) formic acid over 140 minutes followed by 100% (v/v) ACN/0.1% formic acid for 20 minutes and a further 20 minutes of 0% (v/v) acetonitrile/0.1% (v/v) formic acid. A ‘triple play’ mode of analysis was employed, with data-dependent switching between MS and MS/MS, which entailed an initial survey spectrum (MS, 0–10^6^ m/z, zoom scan threshold 200–500 TIC  =  total ion chromatogram) before the three most abundant peptide ions detected were subjected to CID (35% collision energy for 30 ms) and an MS/MS scan (charge state of each ion assigned from the C13 isotope envelope zoom scan). A 500 fmol µl^−1^ solution of glufibrinopeptide (m/z 785.8, [M+2H]^2+^) was used to tune the LTQ. The resulting MS/MS spectra (.raw files) were converted to .dta files using TurboSequest Bioworks version 3.1 (Thermo Fisher Scientific) (parameters  =  threshold cut-off 100, group scan default 100, minimum group count 1, minimum ion count 15, peptide tolerance 1.5). Data were then merged into search-compatible .mgf files which were submitted to Mascot (Matrix Science) for protein identification, searching against a locally-mounted database comprising the *N. caninum* gene predictions release 6 hosted on ToxoDB. Search parameters were as follows: fixed carbamidomethyl modification of cysteine, variable oxidation of methionine, one missed trypsin cleavage, peptide tolerance ±1.5 Da, fragment ion tolerance ±0.8 Da and peptide charge state of +1, +2 and +3.

### Expression of ROP4 in *E. coli*

To confirm that ROP4 was indeed detected by 20D2, we expressed residues 86–509 of the protein in *E. coli*. To obtain the cDNA for protein expression, total RNA was isolated from extracellular *Neospora* parasites by the Trizol method (Invitrogen) as per the manufacturer's instructions and reverse transcription was performed for ROP4 using the primer TAGCCTCGTGTCCTCCGTTTC. This RT reaction was then used as a template for PCR using the same 3′ primer and the 5′ primer CACCCAAGAAGAGGTCGAGCAAGTGC for ROP4. The product was directionally cloned into the pET161 vector which encodes a C-terminal hexahistidine tag for detection and purification [25]. The constructs were sequenced at the ends to verify the coding region, transformed into BL21DE3 strain *E. coli*, and induced for 5 hours with 1 mM IPTG. The pelleted bacteria were lysed in sample buffer and the uninduced and induced lysates were separated by SDS-PAGE, transferred to nitrocellulose, and probed with the mAbs to assess detection of the recombinant protein.

### Analysis of MIC17 proteins from *Neospora* and *Toxoplasma*

The MIC17 protein sequences were obtained from the *Toxoplasma* genome database at http://toxodb.org/toxo/. The proteins correspond to gene models as follows: NcMIC17A (NCLIV_038120), NcMIC17B (NCLIV_038110), NcMIC17C (NCLIV_038100), TgMIC17A (TGME49_000250, TgMIC17B (TGME49_000240), and TgMIC17C (TGME49_000230). The proteins were aligned using CLUSTALW, and colored using the JALVIEW program [37], [38]. The sequences were analyzed for signal peptides using SignalP 3.0 (http://www.cbs.dtu.dk/services/SignalP/), transmembrane domains using TMHMM (http://www.cbs.dtu.dk/services/TMHMM/), and protein domains using PROSITE (http://expasy.org/prosite/) [37], [39], [40].

## Results

### Production of a panel of monoclonal antibodies against purified organelles from *N. caninum*

**Figure 1:**
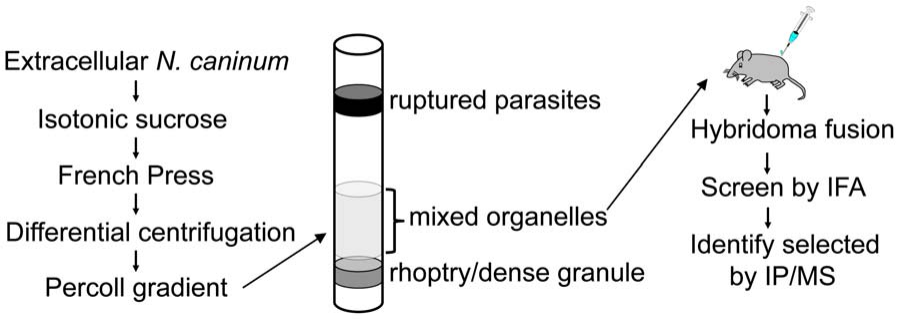
Schematic of organelle purification and analysis using a monoclonal antibody approach. A mixed fraction of organelles was purified from *N. caninum* by disruption of extracellular parasites in the French Press, and isolation of organelles by differential centrifugation and separation on a Percoll gradient. The mixed organellar fraction residing just above the rhoptry/dense granule band was collected and used to immunize a single mouse. Hybridomas were produced and the resulting panel of monoclonal antibodies was screened by IFA. Selected antigens were identified by immunoprecipitation and mass spectrometry.

To identify novel proteins and develop antibody probes for detection of subcellular compartments in *Neospora caninum*, we adapted a protocol for purifying a mixed fraction of organelles from *T. gondii* and isolated a similar fraction from *N. caninim* (Figure 1) [24]. The fractionation consists of disruption of extracellular parasites in the French Press in isotonic sucrose to keep organelles intact, followed by differential centrifugation and isolation on a Percoll density gradient. We chose to collect the fraction positioned just above the rhoptry/dense granule band that contains a mixed fraction of micronemes, rhoptries, dense granules, apicoplasts and mitochondria in *T. gondii* [24]. This mixed organellar fraction was used to immunize a single BALB/c mouse for the production of a panel of monoclonal antibodies (mAbs). Following the fusion, supernatants from hybridoma lines were screened by immunofluorescence assay (IFA) of human foreskin fibroblasts infected with *N. caninum*. To maximize the number of positives obtained, the supernatants were screened using infected cells fixed under two conditions: 100% methanol and 3.7% formaldehyde [25]. The resulting antibodies were further tested by Western blot analysis of *N. caninum* whole cell lysates to determine the approximate size of the antigens recognized (Table 1, the relative molecular mass M_r_ was only reported when an obvious dominant band or bands were identified that had a high likelihood of representing the protein detected by the mAb).

### Antibodies that recognize the parasite surface and inner membrane complex

**Figure 2:**
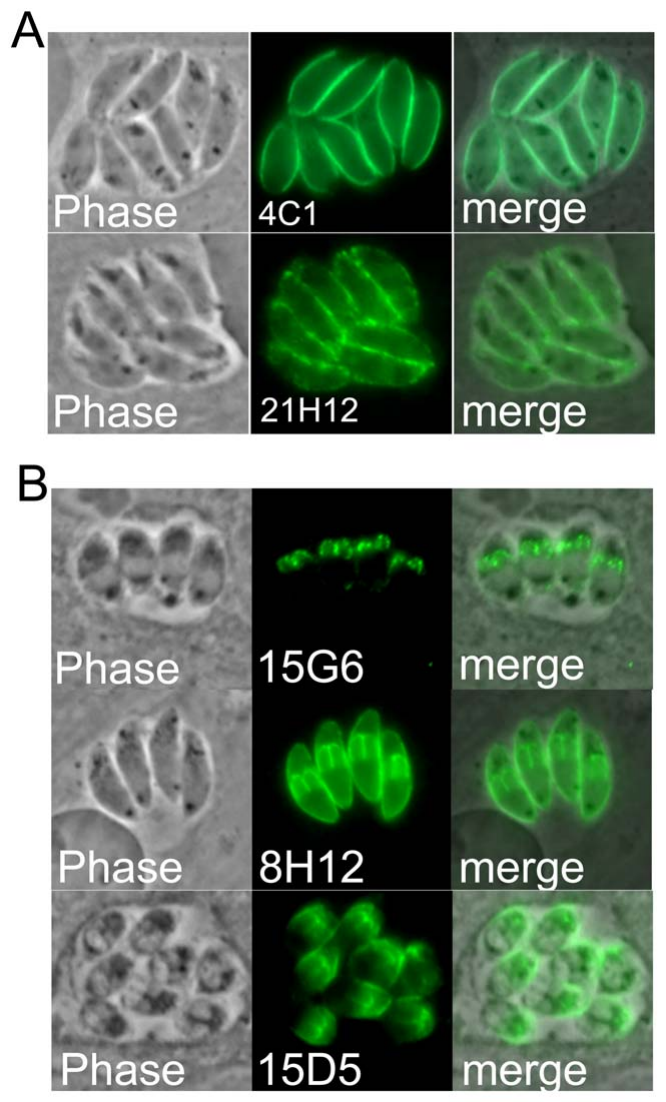
Antibodies detecting the parasite surface and inner membrane complex. A) Phase contrast and fluorescence showing that 4C1 and 21H12 stain the surface of *Neospora*. B) 15G6, 8H12, and 15D5 stain daughter parasites characteristic of the inner membrane complex. 8H12 and 15D5 stain both mother and daughter parasites whereas 15G6 is predominantly detected in daughter cells. 15D5 appears to have a more apical distribution while the others are localized throughout the IMC.

In total, forty-six hybridoma lines were obtained from this single fusion and the antibodies produced were found to stain a variety of cellular compartments in *N. caninum*. Antibodies from two of the hybridomas (4C1, 21H12) stain the parasite surface (Figure 2A). These antibodies are likely detecting highly abundant and immunogenic *Neospora* surface antigens that may be present in the organellar fraction from a minor contamination of the plasma membrane or from surface proteins in transit that co-purified with our organellar fraction. In contrast, three antibodies (15G6, 8H12, and 15D5) stain the surface of developing daughter parasites during the process of endodyogeny, a hallmark of the parasite's inner membrane complex (IMC) (Figure 2B). Each of these displays subtle differences in IMC staining in the parasite. While 8H12 and 15D5 detect antigens that are present in both mother and daughter parasites, 15G6 is predominantly detected in daughter cells. In addition, 15D5 differs in that it appears more concentrated at the apical end of the parasite, whereas the others are localized throughout the IMC. Western blot analysis of *N. caninum* lysates shows that while 8H12 and 15D5 detect low molecular weight antigens, 15G6 detects a >250 kDa protein (Table 1). The previously identified IMC proteins in *T. gondii* migrate considerably faster by SDS-PAGE, indicating that the 15G6 mAb detects a novel IMC protein in *N. caninum* [34], [41], [42], [43], [44].

### Mitochondrial and apicoplast antibodies

**Figure 3:**
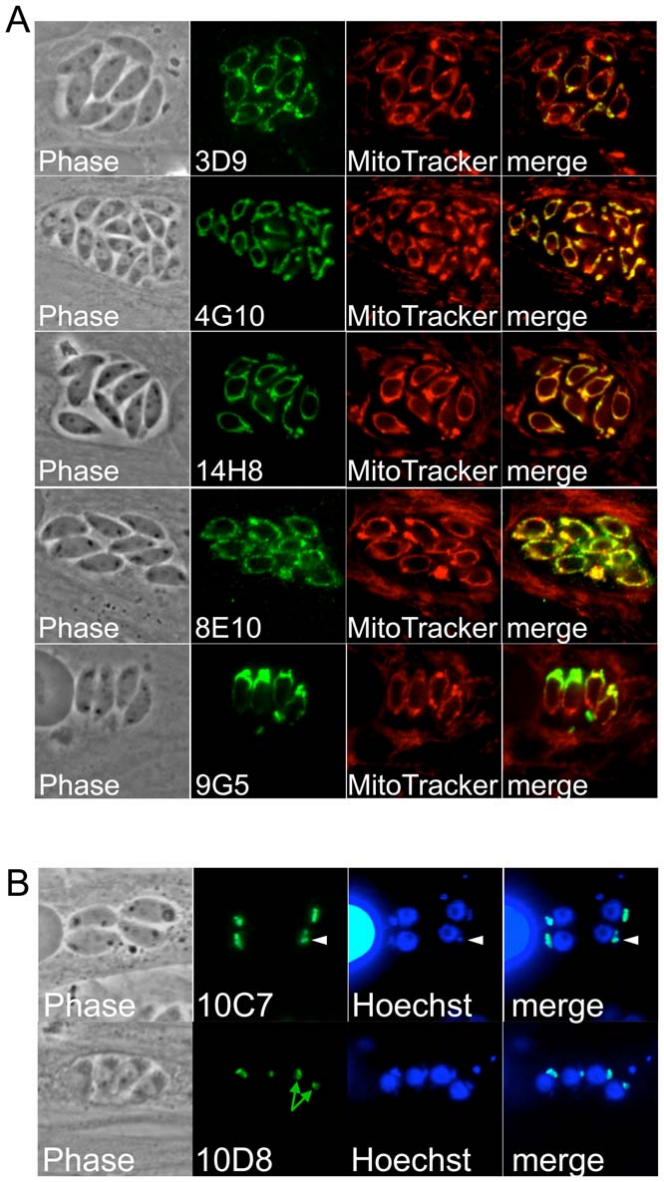
Antibodies detecting the parasite mitochondrion and apicoplast. A) Antibodies detecting the parasite mitochondrion as assessed by Mitotracker colocalization which labels both the host and parasite mitochondria. Each of the monoclonals only stains the single tubular parasite mitochondrion and does not cross-react with the host organelle. B) 10D8 and 10C7 stain the apicoplast as detected by Hoechst co-staining. The apicoplast DNA is seen a single spot that is just anterior to the parasite nucleus (arrowheads). 10D8 shows an example of the central hole that lacks staining corresponding to the matrix of the apicoplast (green arrows).

We also isolated seven hybridoma lines that produced antibodies that stain the apicoplast (2) or mitochondrion (5) in *Neospora* (Figure 3). Like in *Toxoplasma*, the *Neospora* mitochondrion is visualized as a single tubular organelle that often encircles the parasite's nucleus [45], [46]. Mitochondrial localization was confirmed using the mitochondrial probe MitoTracker, which stains both the host and parasite mitochondria dependent on the membrane potential of the organelle (Figure 3A) [29]. All of the anti-mitochondrial antibodies obtained were specific to the parasite and did not cross react with host mitochondria. As expected for the apicoplast, antibodies 10C7 and 10D8 stain a structure just anterior to the parasite's nucleus (Figure 3B) [27], [46]. We confirmed apicoplast localization by costaining with Hoechst stain, which detects the apicoplast genome as a single spot adjacent to the parasite's nucleus (Figure 3B, arrowheads). Similar to that seen in our previous work on the apicoplast protein Atrx1 [27], both antibodies appear to stain the apicoplast membranes as a central hole can often be visualized that lacks staining which corresponds to the lumen of the organelle (Figure 3B, green arrows). 10D8 or 10C7 may in fact recognize the *Neospora* orthologue of *Toxoplasma* Atrx1 as they detect a similarly sized protein by Western blot and were isolated using similar approaches to that used for Atrx1 [27]. To our knowledge, these are the first monoclonal antibodies that detect the apicoplast and mitochondrion in *N. caninum*.

### Antibodies that stain the parasitophorous vacuole and identification of two SRS proteins

**Figure 4:**
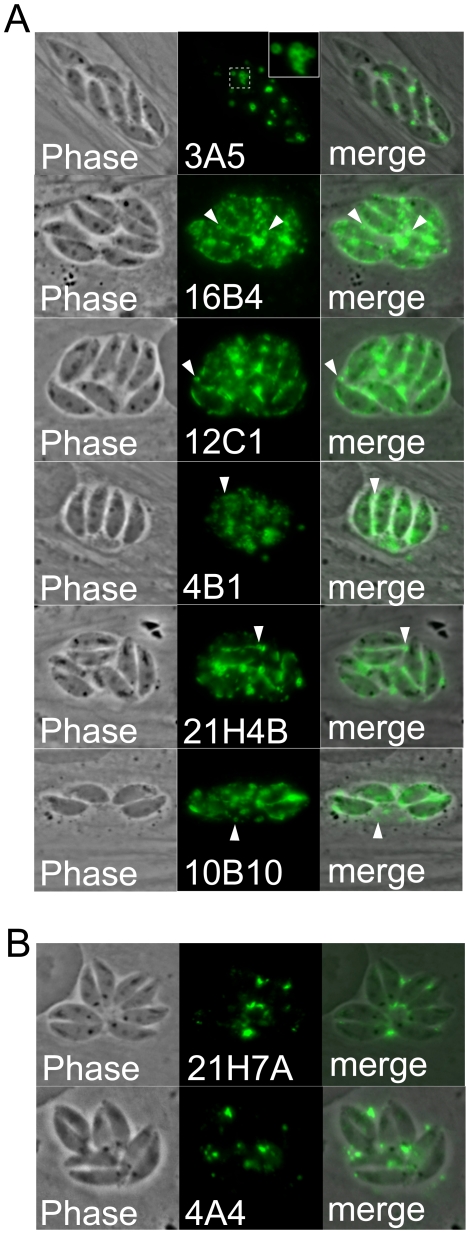
Antibodies that stain the parasitophorous vacuole. A) Phase contrast and IFA of a group of antibodies that appear to stain small circular membraneous blebs within the vacuole (inset and arrowheads) and also have some parasite membrane staining. B) 21H7A and 4A4 stain the parasitophorous vacuole characteristic of dense granule proteins.

The corresponding organellar fraction in *Toxoplasma* is enriched in the parasite's specialized secretory organelles: the rhoptries, micronemes and dense granules. Antibodies from six of the hybridoma lines stain the parasitophorous vacuole (PV) as assessed by phase contrast microscopy (Figure 4A, B). We presumed from previous studies in *Toxoplasma* that vacuole-staining would be indicative of dense granule (GRA) proteins [13], [33]. However, we also noticed that the vacuolar staining in most of these was unusual in that it stained small, round, membraneous blebs in the vacuole and there was also some apparent parasite plasma membrane staining (Figure 4A, inset and arrowheads). To further explore this staining pattern, we immunoaffinity purified two of the proteins from *Neospora* lysates, and identified the proteins by mass spectrometry. We chose 10B10 and 16B4 because they stained relatively low molecular weight proteins (∼45 and 50 kDa respectively) that we suspected could be one of the GRA proteins previously identified in *Toxoplasma* [13], [33]. The immunoaffinity purified proteins were eluted and the eluates separated by SDS-PAGE. In both cases, a clear band corresponding to the expected size of the protein detected by Western blot was obtained (not shown). The bands were excised from the gel, digested with trypsin, and the tryptic peptides identified by mass spectrometry. Surprisingly, both proteins turned out to be related to the SAG family of surface antigens (SRS proteins) present in *Neospora* and *Toxoplasma* (10B10 recognizes NCLIV_010730, 16B4 recognizes NCLIV_068920). The gene model for NCLIV_010730 predicts a C-terminal GPI anchor addition sequence as is typical for this family of proteins [47], [48], [49]. The gene model for NCLIV_068920 is likely incorrect because EST analysis of this gene indicates an extension on the 3′ end of the gene, which then also encodes a predicted GPI anchor addition sequence (not shown). Together, these results suggest that the proteins are lipid anchored to the surface of the parasite or into membraneous structures in the vacuole. The vacuolar localization of these proteins is in stark contrast to the antibodies to SAG-related surface proteins described previously and may indicate that these proteins are shed into the vacuole during intracellular growth. Two other antibodies, 21H7A and 4A4, displayed a more classic staining pattern in the PV and are likely recognizing *bona fide* dense granule proteins (Figure 4B).

### Antibodies that stain the rhoptry body (ROP) and rhoptry neck (RON)

**Figure 5:**
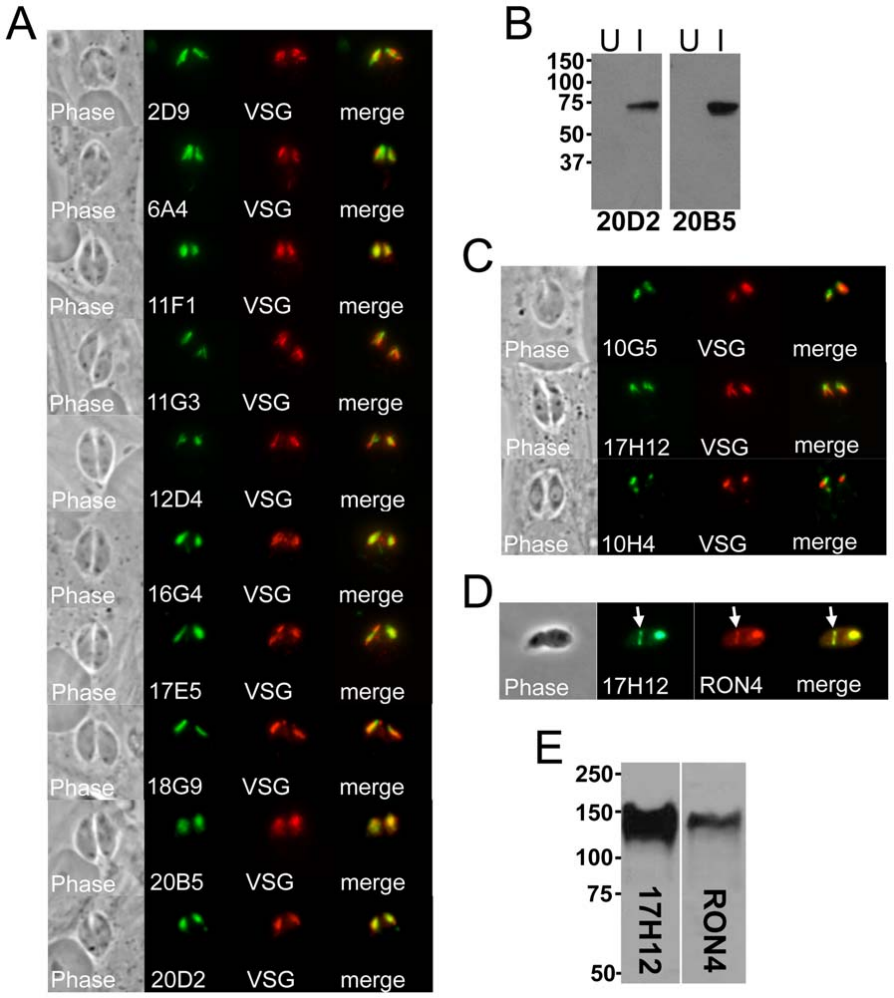
Antibodies against the rhoptry bodies and rhoptry necks. A) Phase contrast and IFA analysis of rhoptry mAbs staining the body portion of the rhoptries. Rhoptry colocalization is shown using a *Toxoplasma* ROP1-VSG construct expressed in *Neospora*. B) mAbs 20D2 and 20B5 detect recombinant ROP4 expressed in *E. coli*. Western blot analysis of the identical strain uninduced (U) and induced (I) for ROP4 expression is shown. C) 17H12, 10G5, and 10H4 stain the more apical neck portion of the organelle and stain slightly apical to that of the ROP1-VSG fusion. A fourth RON protein detected by the mAb 8E3 was previously published [32] and is not shown here. D) 17H12 is secreted into the moving junction (arrow) in partially invaded parasites. The moving junction can be seen as a constriction of the parasite and by colocalization with cross-reactive sera against *Toxoplasma* RON4. E) Western blot analysis showing similar migration for RON4 and 17H12. RON4 is again detected by *Toxoplasma* cross-reactive antibodies against *Neospora* lysates.

Fourteen of the hybridomas secreted antibodies that stain apical, club-shaped structures consistent with the rhoptries (Figure 5A, B) [5], [12]. To confirm rhoptry localization, we transiently transfected *Neospora* with a *Toxoplasma* rhoptry targeting construct (ROP1-VSG) which is a fusion between the rhoptry protein ROP1 and the Trypanosome variant surface glycoprotein (VSG), driven by the ROP1 promoter [31]. The transfected parasites were stained with each of the monoclonal antibodies and colocalization to the rhoptries was verified by anti-VSG staining. We chose to further study two of these antibodies, 20D2 and 2D9, using immunoaffinity chromatography and mass spectrometry to identify their target proteins from *Neospora* lysates as above. 2D9 was found to detect the *Neospora* orthologue of *Toxoplasma* ROP9 and 20D2 identified peptides corresponding to the *Neospora* orthologue of ROP4. In *Toxoplasma*, ROP4 is a member of a large family of closely-related genes containing a kinase domain that are frequently present in multiple copies in the genome [50], [51], [52]. To confirm that 20D2 detected the correct protein identified by mass spectrometry, we amplified residues 86–509 of ROP4 from *Neospora* cDNA, expressed the protein in *E. coli*, and probed Western blots of bacterial lysates expressing the protein with the 20D2 mAb. As seen in Figure 5B, 20D2 detects *E. coli* induced to express the *Neospora* ROP4 gene, but not the same strain of *E. coli* without induction. This result demonstrates that 20D2 does indeed detect *Neospora* ROP4. In addition, we discovered that a second mAb, 20B5, also recognizes ROP4 by screening the antibody against the recombinant protein (Figure 5B).

Four of the anti-rhoptry antibodies (17H12, 10G5, 10H4, and 8E3) stain a more apical region of the organelle that is consistent with the rhoptry necks (Figure 5C, note that in each case the staining is less elongated and more apical relative to VSG). One of these, 8E3, we have previously reported detects the rhoptry neck/moving junction protein RON8 and thus is not shown here [32]. 17H12 also detects a RON protein that is secreted into the moving junction (Figure 5D, arrow) as assessed by IFA of partially invading *Neospora* parasites and costaining with cross-reactive *Toxoplasma* RON4 antisera. 17H12 detects an ∼145 kDa band in *Neospora* lysates that migrates at the same size as *Neospora* RON4, again using cross-reactive *Toxoplasma* RON4 antisera for comparison (Figure 5E). This data suggests that 17H12 may detect RON4 or a similarly sized moving junction protein. Unfortunately, the *Neospora* gene model for RON4 is apparently truncated (NCLIV_030050, positioned at the end of a scaffold), making the determination of whether this antibody stains the recombinant protein as we performed for ROP4 above difficult. Antibody 10G5 detects a 68 kDa protein and 10H4 detects two major bands at 22 and 62 kDa (non-reducing conditions). No rhoptry neck proteins have been reported near these sizes, suggesting that these recognize novel RON proteins. We examined whether these proteins are secreted into the moving junction by staining invading parasites, but could not detect them in the junction (not shown). Together, this group of anti-rhoptry antibodies recognizes several of the central players involved in invasion and host interaction and also likely highlights novel proteins residing in this organelle.

### Anti-micronemal antibodies and identification of MIC17

**Figure 6:**
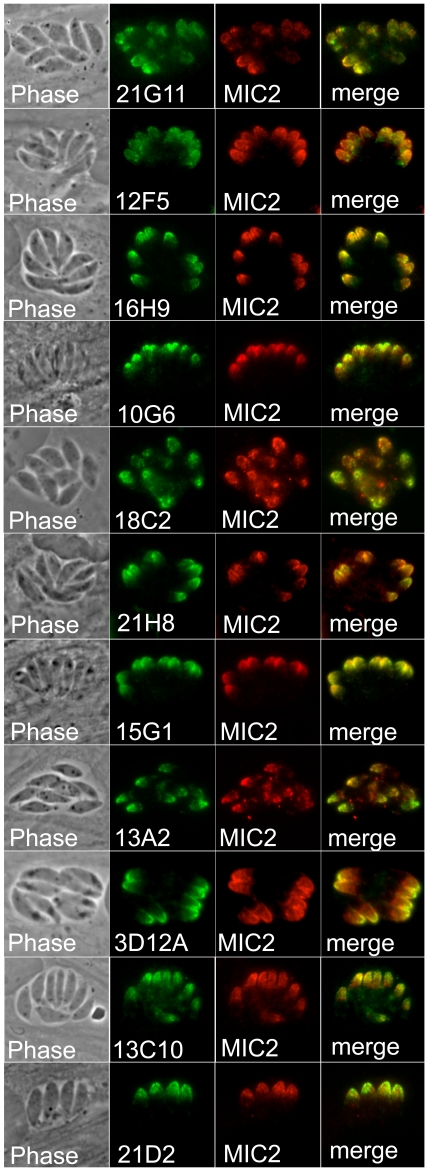
A group of antibodies stain the micronemes. Phase contrast and IFA showing eleven mAbs that stain the apical micronemes of *Neospora*. Colocalization is demonstrated using antibodies against *Neospora* MIC2.

Based on similar fractionations in *Toxoplasma*, we expected the organellar fraction that we purified from *Neospora* to be enriched in micronemes [24]. In agreement with this, we isolated a large number of hybridomas that produced antibodies that stained an apical pattern consistent with the micronemes (Figure 6). Colocalization to the micronemes was confirmed using polyclonal anti-MIC2 antibodies (generously provided by David Sibley). We then selected antibodies 3D12 and 21G11 to identify the corresponding antigens by immunoaffinity chromatography and mass spectrometry as above. 3D12 immunoprecipitated a 72kDa antigen that we identified as the *Neospora* orthologue of apical membrane antigen 1 (AMA1). AMA1 is a micronemal protein identified in *Toxoplasma* and *Plasmodium* that is believed to serve as the parasite plasma membrane anchor for the moving junction complex and is also a vaccine candidate in *Plasmodium* [24], [53].

21G11 immunoprecipitated a 36 kDa protein that corresponds to gene model NCLIV_038110, a novel PAN domain containing protein that is within a group of 3 similar genes tandemly arrayed in the *Neospora* and *Toxoplasma* genomes. This protein was previously identified in the secreted proteome of *Toxoplasma* and in an *in silico* screen for organellar proteins; however, tagged versions of the protein in these studies localized to both the micronemes and the rhoptries, thus preventing a definitive localization [54], [55]. Our results here clearly demonstrate that the NCLIV_038110 protein recognized by 21G11 is a microneme protein which we have thus named MIC17B (we propose that the flanking genes will be MIC17A and MIC17C once microneme localization is confirmed). We further analyzed the MIC17 proteins from both *Neospora* and *Toxoplasma* by aligning the sequences, assessing sorting signals, and conducting protein domain searches (Figure S1). These analyses show that the proteins consist of a secretory signal peptide followed by four tandem PAN domains that contain a conserved set of cysteines in each domain (the second domain contains only five cysteines whereas the others have the more conventional six cysteine residues). The proteins lack predicted transmembrane domains and thus are likely to associate with other membrane-associated *Neospora* MIC proteins to carry out their adhesive functions.

### Detection of unidentified cytoplasmic spots within *N. caninum*

**Figure 7:**
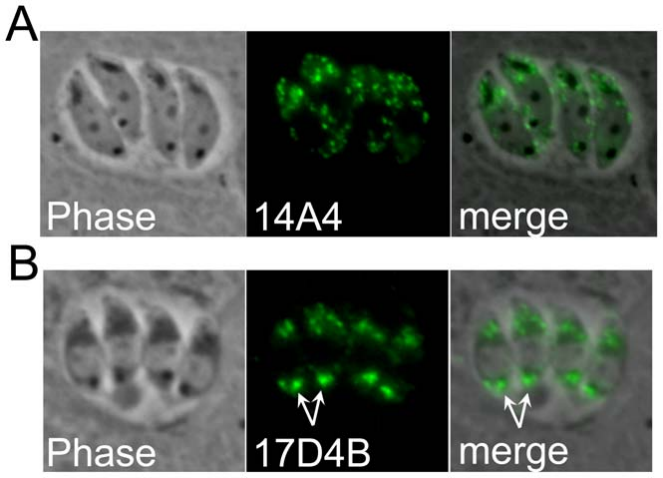
Antibodies staining internal spots in the parasite cytoplasm. A) mAb 14A4 stains a series of internal spots that appear to be generally uniform in size and distributed throughout the cytoplasm. B) mAb 17D4B stains a large posterior spot most intensely (arrows) but also stains a series of 3–4 spots in the apical region of the parasite.

Finally, two antibodies were isolated which stain internal spots within the parasite that we were not able to definitively localize. 14A4 stains a series of spots of relatively uniform size that are distributed throughout the cytosol of the parasite (Figure 7A). This pattern could represent the parasite's dense granules, but we could not locate a suitable marker for directly verifying this. In addition, all previously identified dense granule proteins are secreted into the parasitophorous vacuole [13], thus if 14A4 is recognizing the dense granules, it would likely be staining a resident, rather than a secreted, dense granule protein. 17D4B also stains cytoplasmic spots but with an unusual pattern (Figure 7B). The most intense staining resides in a single larger spot in the posterior end of the parasite. In addition, there is a cluster of 3–4 smaller, less brightly staining spots in the apical portion. To the best of our knowledge, this pattern is unique and may represent a completely novel localization in *N. caninum*.

## Discussion

Our goals of this project were twofold; first, to develop high quality antibody probes to subcellular compartments of *Neospora caninum* and second, to identify novel proteins in this parasite. We were particularly interested in studying the novel organelles unique to apicomplexan parasites, as these would be of interest to a broad array of researchers who study members of the phylum. Our monoclonal antibody approach is timely and ideally suited to *N. caninum* because of the existence of a large number of important intracellular organelles for which few probes are available, an abundance of information regarding compartmentalization in the related apicomplexan *Toxoplasma*, and the recently sequenced *Neospora* genome. By using a mixed fraction of purified organelles as the immunogen, we were able to inject proteins from different subcellular compartments with minimal contamination of immunodominant surface antigens, which likely would have otherwise been overrepresented in the screen at the expense of the hybridomas that we desired. We chose to screen the hybridoma supernatants by IFA instead of the more standard ELISA approach, as we have found in a similar antibody screen in *Toxoplasma* that many antibodies that are functional by IFA do not score positively by ELISA (and vice versa) ([27], Bradley and Boothroyd, unpublished results). The number of positives obtained was increased by screening parasite samples fixed with either formaldehyde or methanol, as many of the antibodies that stained well under one condition worked poorly or not at all using the other. The approach proved to be quite robust and produced such a large number of hybridomas that a number of weaker staining samples were not pursued through the extensive rescreening steps.

While our goal was to identify organellar constituents, we did obtain a significant number of antibodies against parasite surface markers. Some of these neatly stained the periphery of the parasite and probably detect highly immunogenic plasma membrane contaminants of the organellar preparation or proteins *en route* to the parasite surface (Figure 1). Surprisingly, we also found a group of antibodies that stained within the vacuole in a pattern that appears to detect parasite membrane blebs of some sort (Figure 5). Two of these, 10B10 and 16B4, were further studied and each were found to detect a novel SAG-related protein that would have been predicted to be a GPI-anchored protein on the parasite surface. Whether these proteins are secreted into the PV or shed from the parasite surface and whether similar vacuolar localization is seen with these or other SAG-related proteins in *Toxoplasma* remains to be determined (we also cannot exclude the possibility that this localization results from sample fixation, which would best be resolved by expression of fluorescent fusion proteins). We additionally identified three antibodies that detect the IMC, each of which has subtle differences in localization in the IMC in mother and daughter parasites. We and others have recently identified a number of novel IMC proteins in *T. gondii* [34], [41], and it is likely that these antibodies will enable the identification of new players that are important for motility or daughter cell formation, both of which are mediated by this unique structure [34], [41].

We also obtained antibodies specific for the *Neospora* mitochondrion and apicoplast (Figure 3). Whereas almost none of the mAbs recognizing the secretory compartments cross-reacted against *Toxoplasma*, several of these antibodies do cross-react (not shown), indicating that they detect conserved epitopes in the two genera and perhaps reflecting a higher level of conservation of the resident proteins in these compartments. None of the anti-mitochondrial antibodies cross-react with host mitochondria. This may simply be due to more divergent sequences of mitochondrial proteins common to the Eukarya or that these antibodies detect mitochondrial proteins unique to the Apicomplexa. A recent epitope tagging project in *Toxoplasma* revealed a surprising number of novel mitochondrial proteins restricted to the Apicomplexa and close relatives, indicating that fundamental differences exist between the parasite organelle and that of the mammalian host (Li and Morrissette, submitted). The two apicoplast antibodies recognize an ∼60 kDa protein and also appear to localize to the apicoplast membrane, suggesting that they might be detecting the same antigen. If so, they are probably detecting different epitopes as only 10C7 cross-reacts with *Toxoplasma* by IFA (not shown) and they function best under different fixation conditions (Table 1). Together, these mitochondrial and apicoplast probes will be useful tools to study parasite bioenergetics or evaluate the effect of existing and novel therapeutics that target these organelles.

Many of the antibodies isolated stain the rhoptries, secretory organelles that mediate invasion into the host cell, vacuolar formation, and injection of effector proteins into the host cell that modulate host functions [5], [12]. Ten of these stain the rhoptry bodies, including two that we identified as the *Neospora* orthologues of *Toxoplasma* ROP4 and ROP9. The function of ROP9 is unknown, but we suspect it is a fairly immunogenic protein as we isolated several antibodies that detect a similarly sized 36 kDa rhoptry protein. ROP4 is a member of the ROP2 family of proteins that are injected into the host cell where a subgroup of these proteins insert into the cytoplasmic face of the PV membrane using a series of alpha helices in the N-terminal region of the protein [56], [57]. These proteins also contain a kinase domain, but most of these, including ROP4, are predicted to be inactive pseudokinases because they lack conserved residues required for activity [51], [52]. Because this is a large group of related proteins that are often present in multiple copies that have resulted in incorrect annotation in *Toxoplasma*, we verified that the 20D2 antibody recognizes recombinant *Neospora* ROP4 expressed in *E. coli*. This data further indicates that we are identifying the correct protein even under the most challenging conditions (e.g. large families of similar proteins) and enabled us to show that 20B5 also detects this protein.

Four of the rhoptry mAbs detected RON proteins, two of which are secreted into the moving junction (one of which we previously reported as RON8 [32]). The 17H12 antibody may recognize *Neospora* RON4 due to its similar migration by Western blot analysis, but the RON4 gene model for *Neospora* is likely not complete and we have not been able to obtain the full length cDNA to verify this. Intriguingly, whereas anti-RON4 antibodies in *Toxoplasma* recognize the rhoptry necks and moving junction, they also detect RON4 within the vacuole [25], [58]. No vacuolar staining is seen in *Neospora* with 17H12 or with cross-reactive polyclonal antibodies against *Toxoplasma* RON4 (data not shown), indicating that RON4 localization within the vacuole is specific to *Toxoplasma* and not seen in *Neospora*. In addition, *Neospora* RON4 also migrates substantially higher than its counterpart in *Toxoplasma*, which may reflect differences in proteolytic processing of this protein between these organisms [32]. The other two RON antibodies likely stain novel proteins and will help to understand how the non-junction RON proteins modulate host-pathogen interactions in *Neospora* and other apicomplexans.

Another large group of antibodies produced stain the micronemes in *N. caninum*. Western blot analysis indicates that these antibodies detect a range of different proteins in *Neospora*. Both of the antibodies that we chose to characterize further turned out to be particularly interesting. 3D12A recognizes AMA1, which is believed to anchor the invading parasite to the secreted RON proteins in the moving junction complex [11]. In our 3D12A immunoprecipitation, we did see a series of faint bands migrating at 120–140 kDa in addition to the 72 kDa target band that are consistent with the moving junction proteins RON2/4/5. The low abundance of these co-precipitating bands compared to that seen in immunoprecipitations of the RON complex in AMA1 pulldowns in *Toxoplasma* likely reflects the more stringent RIPA conditions used here compared to the lower salt and detergents used to isolate the moving junction complex in previous studies in *Toxoplasma* [32], [58], [59]. It will be interesting to determine if 3D12A is able to block *Neospora* invasion as has been previously seen in *Neospora*, *Toxoplasma* and *Plasmodium* [60], [61].

The second antibody, 21G11, detects a novel microneme protein which we termed MIC17B, whose localization was not previously known in *Neospora* or *Toxoplasma*. The four PAN domains present in the protein indicates a role in adhesion to host cells similar to other microneme proteins [10]. The MIC17B proteins lack a transmembrane domain and thus may be “escorted” to the parasite surface as a protein complex with other MIC proteins, as has been seen for the MIC1/4/6 complex [62]. While we did not see obvious co-precipitating proteins during immunoaffinity purification of MIC17B, as described above this is expected due to the RIPA detergents used for purifying the target protein. Isolation of the protein under less stringent detergent conditions will likely resolve whether the MIC17 proteins partner with escorter proteins for their delivery to the micronemes and subsequent adhesive functions following secretion. The identification of MIC17 highlights another advantage of the monoclonal antibody approach in that while tagging of proteins is often convenient, the tag can alter the localization of proteins. This appears to be the case here because epitope-tagged MIC17B in *Toxoplasma* localizes partly to the rhoptries and partly to the micronemes, precluding a definitive demonstration of compartmentalization [54], [55]. In addition, the monoclonal antibody developed here can be readily used for the study of this protein in a wide number of strains without the labor of creating tagged versions of the proteins.

The final group of antibodies stains small spots within the cytoplasm of the parasite. While 14A4 could be simply detecting a resident dense granule protein, 17D4B stains a unique pattern in *Neospora* consisting of a large posterior spot and additional smaller anterior spots. In *Toxoplasma*, a newly discovered plant-like vacuole shares some similarities in that it stains a large vacuole [63]. However, the *Toxoplasma* plant-like vacuole is typically anterior, and breaks up into smaller vacuoles in intracellular parasites unlike the staining pattern observed with 17D4B. The identification of the proteins recognized by these antibodies will help to determine their precise localization and enable comparisons with other apicomplexans.

In summary, we have developed a wide array of highly specific and robust probes for studying the cell biology of *N. caninum*, especially the crucial processes of cell attachment and invasion mediated by the parasite's specialized secretory organelles. Many of these antibodies detect important proteins previously identified in *Toxoplasma* and verify their compartmentalization in *N. caninum*. Other antibodies have revealed the localization for proteins that have not been previously studied. As most of the probes we have identified here do not cross-react with *Toxoplasma*, an additional utility is the potential use of these *Neospora* proteins as tagged copies for study in *Toxoplasma*, as we suspect the orthologues would generally traffic correctly and often functionally mimic the *Toxoplasma* protein. In addition, the monoclonal antibody probes identified here may prove useful for diagnostic purposes to distinguish between *Neospora* and other closely related parasites including *T. gondii*, *N. hugesi* and *H. heydorni*.

## Supporting Information

Figure S1 **Alignment of MIC17A-C from** ***Neospora*** **and** ***Toxoplasma*** **.** The predicted protein sequences for MIC17 proteins were obtained from the *Toxoplasma* genome (http://toxodb.org/toxo/). The alignment shows sequence identity in dark blue and similarity in light blue. The four predicted PAN domains are underlined in red and the conserved cysteines common to PAN domains are shown in yellow. Note that the second PAN domain contains five conserved cysteines instead the more conventional six cysteines.
(TIF)
