# Supplementary figures and images for: Identification of Novel Proteins in Neospora caninum Using an Organelle Purification and Monoclonal Antibody Approach
Source: PLoS One. 2011 Apr 4;6(4):e18383. doi: 10.1371/journal.pone.0018383 (PMC3070720; doi:10.1371/journal.pone.0018383)

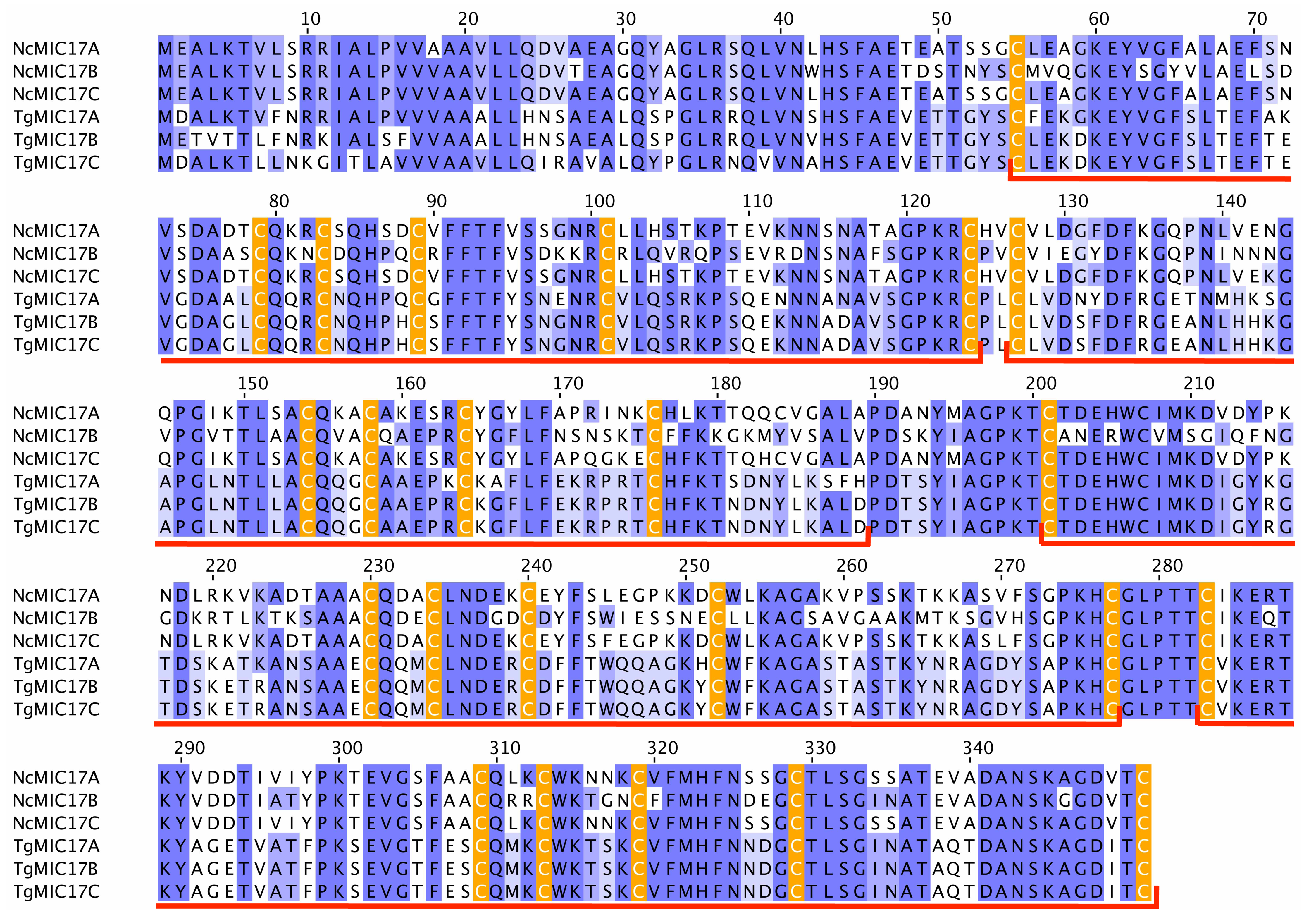

Supplement: Figure S1 — Alignment of MIC17A-C from Neospora and Toxoplasma . The predicted protein sequences for MIC17 proteins were obtained from the Toxoplasma genome (http://toxodb.org/toxo/). The alignment shows sequence identity in dark blue and similarity in light blue. The four predicted PAN domains are underlined in red and the conserved cysteines common to PAN domains are shown in yellow. Note that the second PAN domain contains five conserved cysteines instead the more conventional six cysteines. (TIF) [file pone.0018383.s001.tif]
